# *Sarcodon aspratus* Polysaccharide Ameliorates Type 2 Diabetes Mellitus Symptoms by Regulating Intestinal Barrier and Intestinal Microbiota

**DOI:** 10.3390/foods14223871

**Published:** 2025-11-12

**Authors:** Dongjing Zhang, Xiuying Sun, Haichao Wang, Lei Chen

**Affiliations:** 1 School of Biological and Food Engineering, Suzhou University, Suzhou 234000, China; zhangdongjing1987@163.com (D.Z.); 14755080928@163.com (X.S.); wanghaichao@ahszu.edu.cn (H.W.); 2 Anhui Key Laboratory of Ecological Engineering and Biotechnology, School of Life Sciences and Medical Engineering, Anhui University, Hefei 230601, China

**Keywords:** *Sarcodon aspratus* polysaccharides, T2DM mice, intestinal barrier, intestinal microbiota, AMPK signaling pathway

## Abstract

*Sarcodon aspratus* fruiting polysaccharides (SAFP) exhibit multiple therapeutic properties. In this study, a type 2 diabetes mellitus (T2DM) mouse model was established using a high-fat diet (HFD) and streptozotocin to evaluate the antidiabetic potential of SAFP. Then the benefits of SAFP on glucolipid metabolism, gut barrier integrity and intestinal microbiota were evaluated. The results indicated that SAFP alleviated disturbances in glycolipid metabolism and insulin resistance through activating Adenosine 5′-monophosphate (AMP)-activated protein kinase (AMPK) signaling pathway. Furthermore, SAFP ameliorated hepatic inflammation and hepatic steatosis, as well as restored dysbiosis in hepatic function. Notably, SAFP enhanced intestinal mucosal architecture and strengthened epithelial barrier functionality through upregulated expression of tight junction components such as Zonula occludens-1(ZO-1), Claudin-1, and Occludin proteins. The 16S rRNA analysis indicated that SAFP has the potential to restore the intestinal microbial barrier in T2DM mice through elevation of short-chain fatty acids (SCFAs) concentrations and regulation of microbial community imbalances. This research offers foundational evidence supporting the utilization of SAFP as an innovative dietary supplement or prospective prebiotic component in functional food formulations targeting diabetes management.

## 1. Introduction

Diabetes mellitus (DM), a group of long-term metabolic conditions marked by sustained high blood sugar levels, poses a major challenge to worldwide public health. DM encompasses type 1 diabetes (T1DM), type 2 diabetes mellitus (T2DM), gestational diabetes, and less common variants [1]. Notably, nearly 90% of diabetes cases involve T2DM, which arises from multiple pathological mechanisms such as insulin resistance, oxidative damage, and chronic inflammatory processes [2].

As a chronic low-grade inflammatory disease, the pathogenesis of T2DM may be linked to dysfunction of the intestinal mucosal barrier or dysbiosis within the intestinal microbiota [3]. The intestinal mucosal barrier comprises mechanical, chemical, microbial, and immune components that are crucial for protecting the host against antigens, bacteria, and viruses. This multifaceted barrier plays a significant role in maintaining overall health [4,5]. As the primary selective barrier system unique to the intestine, the intestinal mechanical barrier plays a vital role in safeguarding the body against damage caused by exogenous antigens and maintaining homeostasis within the intestinal environment [6,7]. The intestinal microbiota plays a crucial role not only in food digestion, nutrient absorption, and energy supply but also in regulating the normal physiological functions of the human body [8]. Furthermore, it plays a crucial role in the progression of obesity and T2DM by modulating the function of the gut endothelial barrier [9], SCFAs, and chronic inflammation induced by metabolic endotoxemia. The diversity of the intestinal microbiota in patients with type 2 diabetes is significantly lower than that observed in healthy individuals. Notably, the abundances of *Bifidobacterium*, *Lactobacillus*, *Akkermansia*, and *Prevotella* are markedly diminished. Furthermore, there is a reduction in the abundances of *Verrucomicrobia*, *Bacteroidetes*, and *Actinobacteria* as well [10]. Thus, adjusting the microbial profile in individuals with T2DM, such as reducing certain harmful bacteria while promoting specific beneficial bacteria through dietary interventions may be a key strategy for the treatment of T2DM [11]. Metabolic syndrome is a group of clinical syndromes characterized by obesity, hyperglycemia, dyslipidemia, and hypertension, which seriously increases the risk of type 2 diabetes and affects the health of the body [2]. Throughout the years, the strategies for treating metabolic syndrome have always focused on improving insulin sensitivity. The prolonged administration of insulin-sensitizing agents, such as metformin and rosiglitazone, is frequently associated with gastrointestinal adverse effects, which can lead to suboptimal patient adherence [12].

Therefore, there is growing research focus on seeking safe and effective natural bioactive compounds that can reduce the risk of T2DM without causing side effects. Polysaccharides, the main bioactive compounds of edible and medicinal fungi, have attracted significant academic attention because of their prominent functions on intestinal health and glucolipid metabolism [13,14,15]. As a precious wild edible fungus, *Sarcodon aspratus* mainly grows in Yunnan province and Sichuan province in China; it is popular for its taste and health values. Emerging research has demonstrated polysaccharide extracted from *Sarcodon aspratus* exhibited multiple biological activities, including immune response enhancement [16], detoxification against 5-fluorouracil toxicity in murine tumor models [17], and regulation of lipid homeostasis and glucose tolerance in HFD-fed mice [13]. Prior research has demonstrated that *Sarcodon aspratus* fruiting polysaccharides (SAFP) can mitigate bleomycin-induced pulmonary fibrosis by ameliorating oxidative stress and inflammatory response through the NF-κB/TGF-β1/MAPK pathway [18]. Additionally, our prior investigations revealed that SAFP ameliorated WIRS-induced gastric ulcers through TLR4/Nrf2 pathway modulation while restoring gut microbial balance [19]. Nevertheless, to date, it remains unclear whether SAFP exerts any beneficial effects on T2DM mice. Therefore, this study aims to evaluate the regulatory effects of SAFP on glycolipid metabolism and insulin resistance in diabetic murine models, while concurrently assessing its influence on intestinal mucosal integrity and microbial community equilibrium. Furthermore, we aim to investigate the molecular mechanisms involved in these physiological interactions.

## 2. Materials and Methods

### 2.1. Materials and Reagents

The *Sarcodon aspratus* fruiting polysaccharides (SAFP) were prepared by the water extraction and alcohol precipitation method, according to the method described in the previous study [19]. All primary and secondary antibodies were obtained from Abcam (Cambridge, UK). All other reagents used in this experiment are of analytical grade and were sourced from local reagent manufacturers.

### 2.2. Animals and Treatment

**Figure 1:**
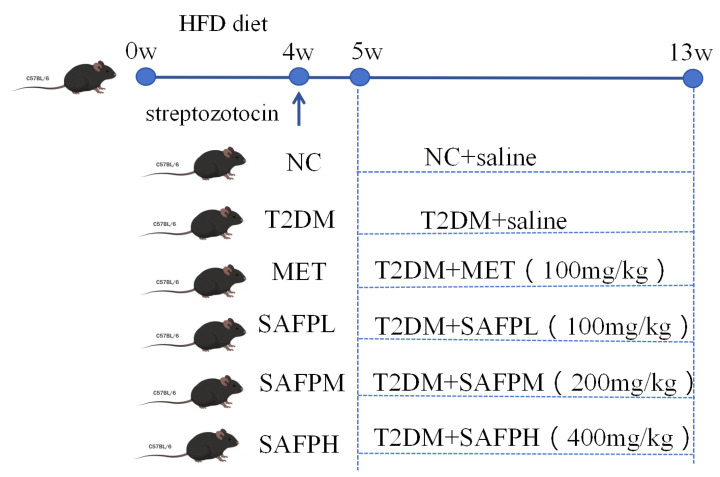
Animal experiment protocol and design.

Healthy male BALB/c mice, weighing 18 ± 5 g (6 to 8 weeks old), were procured from the Animal Center of Anhui Medical University in Hefei, China (animal license No. SCXK (Wan) 2017-001). Both the standard laboratory rodent diet and high-fat diet were also procured from the same source. Experimental protocols from the Laboratory Animal Welfare and Ethics Guidelines of China (GB/T35892-2018) were followed [20], with ethical approval from Anhui University’s IACUC (No. IACUC(AHU)-2023-061) and complying with ARRIVE reporting standards [21]. Mice were maintained under controlled environmental conditions (25 ± 2 °C ambient temperature, 55 ± 5% relative humidity) with standardized 12 h/12 h light-dark cycling. The type 2 diabetes mellitus model was developed using a revised methodology from prior studies [22]. The animal experiment protocol and design were shown in Figure 1. Following a 7-day acclimatization period, the healthy mice were divided randomly into two main experimental cohorts (i) Normal control group (standard laboratory rodent diet: 60% carbohydrate, 20% protein, 10% lipids, 8% dietary fiber, 10% vitamin/mineral substance, 8 mice) and (ii) HFD group (high-fat diet: 10% lard, 15% sugar, 1% cholesterol, 0.5% sodium cholate, 0.2% methylthiouracil, and 73.3% standard laboratory rodent diet; 40 mice). After a duration of four weeks, the mice in the HFD group received intraperitoneal injections of streptozotocin at a dosage of 50 mg/kg, administered four times within one week. In contrast, the mice in the normal control (NC) group were injected with citrate buffer and served as normal controls. On the third day following the fourth injection, mice exhibiting fasting blood glucose (FBG) levels exceeding 11.1 mmol/L were classified as T2DM mice. We conducted the pre-experiment to determine the doses of SAFP in experimental groups before we started the formal experiment. The confirmed diabetic mice (*n* = 40) were subsequently stratified into five groups (*n* = 8/group): (I) T2DM group (T2DM, administered daily with saline solution); (II) MET group (T2DM + 100 mg/kg metformin (MET) via intragastric gavage); (III) SAFPL group (T2DM + 100 mg/kg SAFP administered daily via intragastric gavage); (IV) SAFPM group (T2DM + 200 mg/kg SAFP administered daily via intragastric gavage); and (V) SAFPH group (T2DM + 400 mg/kg SAFP administered daily via intragastric gavage). SAFP treatment lasted for 8 weeks. Weekly assessments of murine body mass and fasting blood glucose (FBG) levels were conducted. The blood was collected via the retro-orbital venous plexus of the mouse to obtain serum, facilitating the measurement of blood glucose levels at regular intervals. Following the sacrifice of the mice, blood acquisition via cardiac puncture yielded serum through centrifugation (3000 rpm, 20 min) for biochemical evaluation. Post-euthanasia, hepatic tissues were immediately excised and measured to calculate hepatic index (liver mass (mg)/body weight (g)), with aliquots preserved for histopathological examination and biochemical assays. Concurrently, ileal tissues were harvested for supplementary investigations. All biological specimens were cryopreserved at −80 °C pending subsequent analytical procedures.

### 2.3. Assessment of Glucose Tolerance and Insulin Sensitivity

The oral glucose tolerance test (OGTT) was conducted to evaluate the sensitivity of mice that had been fasted for 12 h to a glucose overload. Mice that had been fasted overnight were administered a gavage of 3 g/kg glucose, and their blood glucose levels were measured using a hand-held glucometer (Accu-Chek, Roche, Basel, Switzerland). Blood samples were obtained via the tail vein at time points of 0, 15, 30, 60, and 120 min. The area under the blood glucose curve (AUC) was subsequently calculated using established methodologies [22]. Fasting serum insulin levels (FINS) were measured using a commercial ELISA kit [23]. The Homeostasis Model Assessment (HOMA)-insulin resistance (IR) and insulin sensitivity index (ISI) were determined according to validated computational models referenced in prior research [24].

### 2.4. Analysis of Biochemical Parameters in Serum and Liver

Total cholesterol (TC, BES2052CMT), triglyceride (TG, BES-BK2761B), high density lipoprotein cholesterol (HDL-C, BES-BK2771B), low density lipoprotein cholesterol (LDL-C, BES2054CMT), alanine aminotransferase (ALT, BES1368K) and aspartate aminotransferase (AST, BES2425K) in serum were determined using commercial detection kits (Beijing Boosen Biotechnology Co., LTD, Beijing, China) according to the manufacturer’s instructions. The levels of T-AOC, T-SOD, tumor necrosis factor-α (TNF-α), interleukin-1β (IL-1β) and interleukin-6 (IL-6) in hepatic tissue were measured according to the protocols provided by the respective assay kits from Beijing Boosen Biotechnology Co., Ltd. (Beijing, China).

### 2.5. Histopathological Investigations

Liver specimens were immediately fixed in 4% paraformaldehyde solution, then dehydrated through graded ethanol and embedded in paraffin blocks. Subsequently, the tissues were sectioned into 5 μm slices and stained with hematoxylin and eosin (H&E) as well as Masson’s trichrome reagents using standard techniques [25]. For lipid visualization, freshly harvested hepatic samples underwent cryopreservation before being treated with oil-red O solution (Sigma-Aldrich, St. Louis, MO, USA) for 10 min intervals. Glycogen distribution patterns were evaluated through Periodic acid-Schiff (PAS) staining (Sigma-Aldrich, St. Louis, MO, USA) to microscopically assess the severity of hepatic injury. After the mice were sacrificed, an incision was made along the abdominal wall to expose the connection between the stomach and intestine. The surrounding tissues were carefully separated using tweezers. The entire digestive tract, extending from the end of the esophagus to the rectum, was excised from the abdominal cavity and transferred to a culture dish containing physiological saline. The intestines were rinsed in physiological saline to eliminate blood stains and residual materials. They were then laid flat in a Petri dish for clear observation of their complete anatomical structure: stomach, small intestinal tissue (duodenum, jejunum, and ileum), cecum, colon, and rectum. Then the jejunum tissue was cut at corresponding locations. Concurrently, fresh jejunum tissue that had been immersed in 4% of paraformaldehyde solution were similarly stained with H&E. Histopathological imaging was conducted using an Olympus IX73-DP80 microscopy system (Olympus Corp., Tokyo, Japan). A total of four randomized microscopic fields per specimen were randomly selected for analysis and subsequently averaged.

### 2.6. Immunohistochemistry (IHC) Analysis

IHC assay was carried out as per our previously described method [22]. The jejunum tissue samples were fixed in freshly prepared 10% neutral-buffered formalin, embedded in paraffin wax blocks, and subsequently sectioned into slices of 5 μm thickness. After deparaffinization and rehydration, tissue sections were exposed to hydrogen peroxide solution (3% *v*/*v*) to block endogenous peroxidase activity. Next, slides were treated overnight at 4 °C with primary antibodies (Abcam, Cambridge, UK) against ZO-1 (ab307799), Occludin (ab216327) and Claudin-1 (ab211737) at 4 °C overnight. Next, the jejunum tissue slices were incubated with secondary antibodies (anti-rabbit IgG) and subsequently stained using diaminobenzidine and hematoxylin. Digital imaging was conducted with an IX73 Olympus microscope coupled with a DP80 dual CCD camera (Olympus Corp., Tokyo, Japan). A quantitative analysis of immunopositive cells was performed across five randomly selected microscopic fields per sample using IPP image analysis software (Image-Pro Plus 6.0).

### 2.7. Western Blotting

Liver tissues were processed into homogenates using RIPA lysis buffer (containing 1 mM PMSF) supplemented with protease inhibitor cocktail (Sigma-Aldrich, St. Louis, MO, USA). Following centrifugation at 12,000× *g* for 15 min, the resultant supernatants underwent protein concentration determination through a bicinchoninic acid assay kit (Nanjing Jian Cheng Bioengineering Institute, Nanjing, China). Equal protein aliquots were subjected to electrophoresis on 10% SDS-PAGE gels followed by electrotransfer onto PVDF membranes. After blocking with 5% non-fat milk dissolved in tris-buffered saline Tween-20 (TBST) solution, the membranes were probed with primary antibodies (Abcam, Cambridge, UK) targeting key metabolic regulators: AMPKα (ab32047), p-AMPKα (AA393), G6Pase (ab93857), PEPCK (ABC1691), and β-actin (GB15001) through overnight incubation at 4 °C. Following three TBST washing cycles, the membranes were exposed to HRP-conjugated IgG secondary antibodies (Abcam, Cambridge, UK). The protein bands were visualized with enhanced chemiluminescence detection reagents (Sigma-Aldrich, St. Louis, MO, USA) and imaged with a gel imaging system (Tanon Technology, Shanghai, China). The optical density quantification of relevant bands was measured through Labworks gel imaging and software (Tanon 5200 Multi, Labworks LLC, Lehi, UT, USA).

### 2.8. SCFAs Assay

Standard solutions of acetic, propionic, butyric, and valeric acids were prepared in diethyl ether at six concentration levels: 1, 5, 25, 100, 250, and 500 μg/mL. For sample preparation, 500 mg of cecal material was homogenized with 1 mL deionized water and centrifuged at 5000 rpm (10 min) to obtain clarified liquid. The aqueous phase underwent acidification with 20 μL 10% sulfuric acid prior to ether extraction. Quantitative analysis of short-chain fatty acids (SCFAs) was performed using an Agilent GC7890 system (Agilent Co., Santa Clara, CA, USA) featuring a TG-WAXMS capillary column (30 m × 0.25 mm × 0.25 μm). Chromatographic separation employed an initial 80 °C hold (1 min), followed by a 10 °C/min ramp to 220 °C with final isothermal maintenance for 5 min. These concentrations were calculated based on a standard calibration curve.

### 2.9. Analysis of Intestinal Microbiota

Microbial community genomic DNA from cecal contents was extracted using an E.Z.N.A. ^®^Stool DNA Kit (Omega D4015, Bio-Tek Inc., Norcross, GA, USA) according to the manufacturer’s instructions. The amplification of V3–V4 regions of 16S rDNA genes was performed using bacterial universal primer set, and samples were sequenced on an Illumina MiSeq PE300 platform (LC-Bio Technology Co., Ltd., Hangzhou, China) and analyzed according to the manufacturer’s instructions. Post-sequencing, high-quality sequences underwent filtration and were subsequently grouped into operational taxonomic units (OTUs) at 97% sequence similarity using the Greengenes reference database. Within-sample diversity metrics (Chao 1, observed species, and Shannon indices) were computed to evaluate alpha diversity. Inter-group differences in microbial composition (beta-diversity) were visualized through principal coordinate analysis (PCoA) diagrams.

### 2.10. Statistical Analysis

The final data represent the outcomes of three independent experiments and are presented as the mean ± standard deviation (SD). Group comparisons were conducted through one-way ANOVA with Tukey’s post hoc analysis. The analysis of variance was performed using SPSS version 21.0.0.0 software to identify significant differences (*p* < 0.05). In figure captions, different letters indicate statistically significant differences at the *p* < 0.05 level.

## 3. Results

### 3.1. SAFP Intervention Alleviates Metabolic Disturbance in T2DM Mice

**Figure 2:**
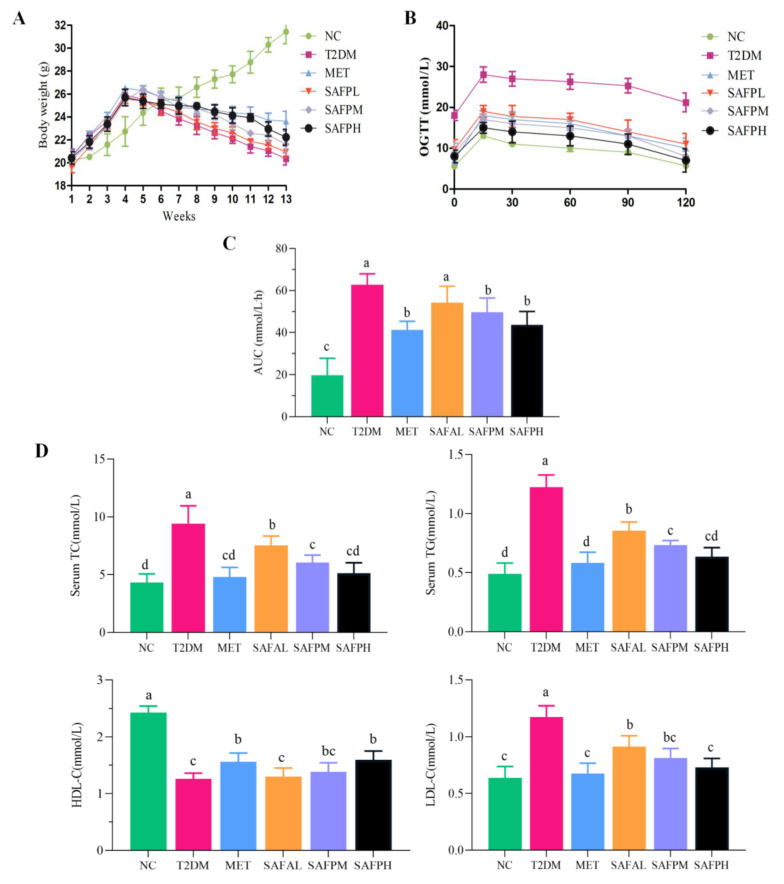
SAFP intervention alleviates metabolic disturbance in T2DM mice. (**A**) Body weight. (**B**) OGTT level. (**C**) AUC level. (**D**) Serum TC, TG, HDL-C, and LDL-C. Values are represented as mean ± SD (*n* = 8). Different small letters indicate that values are significantly different at the *p* < 0.05 level.

Metabolic disturbances typically encompass glycometabolism and lipometabolic disturbance, which are commonly observed in the clinical manifestations of T2DM [26]. As manifested in Figure 2A, experimental groups exhibited substantial elevation in body mass compared to normal control (NC) animals before streptozotocin administration. However, the body weights of mice observably decreased in the T2DM group, which were significantly reversed by supplementation with MET or SAFP. The effect of SAFP on glycometabolism homeostasis was evaluated by OGTT. Figure 2B,C illustrated that T2DM mice showed poorer ability in terms of glucose tolerance and thus the glucose content declined slowly after 16 min when compared to the NC group, whereas MET or SAFP intervention led to a rapid decrease in glucose content. Additionally, the elevated area under the curve (AUC) observed in T2DM mice was remarkedly ameliorated by SAFP intervention. These findings suggested that SAFP supplementation effectively ameliorated impaired glucose tolerance in T2DM mice. To investigate the therapeutic potential of MET and SAFP on lipid regulation in diabetic models, serum concentrations of key atherogenic (TG, TC, LDL-C) and anti-atherogenic (HDL-C) biomarkers were analyzed. As shown in Figure 2D, the T2DM group exhibited a significant increase in serum levels of TC, TG, and LDL-C alongside a decrease in HDL-C compared to the NC group. Notably, administration of SAFP resulted in a marked elevation of anti-atherogenic lipid indices while concurrently reducing atherogenic lipid indices in a dose-dependent manner. These findings provide compelling evidence that SAFP administration mitigated body weight loss, as well as mitigated the hyperglycaemia and hyperlipidemia, improving glycolipid metabolism homeostasis.

### 3.2. SAFP Intervention Alleviates Insulin Resistance in T2DM Mice

**Figure 3:**
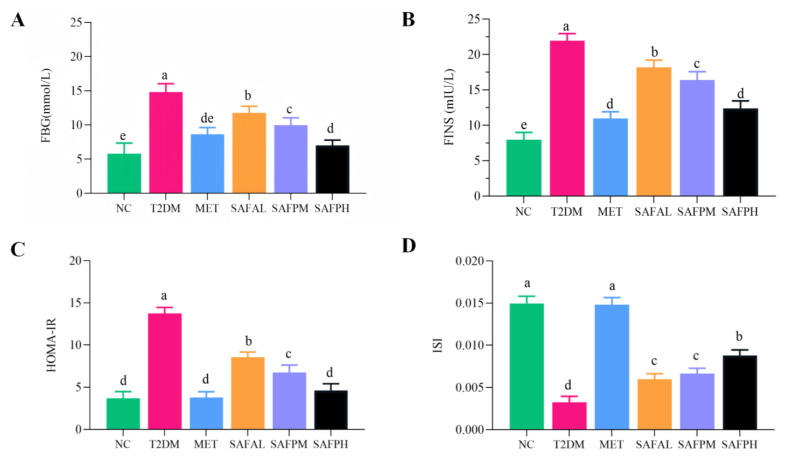
SAFP intervention alleviates insulin resistance in T2DM mice. The levels of FBG (**A**), FINS (**B**), HOMA-IR (**C**) and ISI (**D**) were quantitatively assessed. Values are expressed as mean ± SD (*n* = 8). Different small letters indicate that values are significantly different at the *p* < 0.05 level.

To investigate the effects of SAFP administration on insulin sensitivity and resistance, we measured FBG and FINS and calculated the HOMA-IR index and ISI. As shown in Figure 3, FBG and FINS were markedly elevated in T2DM mice, but these levels were reduced following treatment with SAFP or MET. As a result, a significant increase in HOMA-IR and a decrease in ISI were observed in the T2DM mice. Importantly, both SAFP and MET interventions led to a notable reduction in HOMA-IR while simultaneously enhancing ISI when compared to the T2DM group. These findings suggested that SAFP administration enhanced insulin sensitivity and alleviated insulin resistance in diabetic mice in a dose-dependent manner. Strikingly, the therapeutic outcomes achieved with 400 mg/kg SAFP dosage showed comparable effectiveness to MET intervention protocols.

### 3.3. SAFP Intervention Alleviates Hepatic Steatosis and Dysfunction

**Figure 4:**
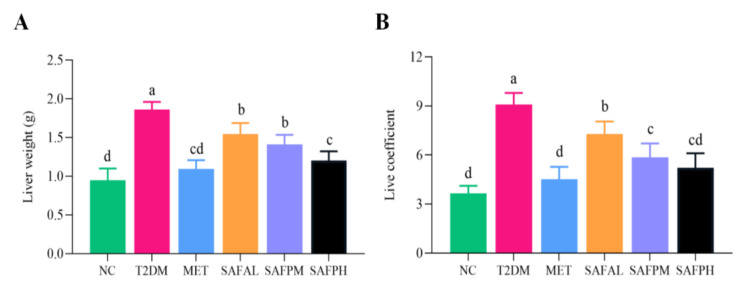
(**A**) Live weight. (**B**) Liver index. Values are expressed as mean ± SD (*n* = 8). Different small letters indicate that values are significantly different at the *p* < 0.05 level.

**Figure 5:**
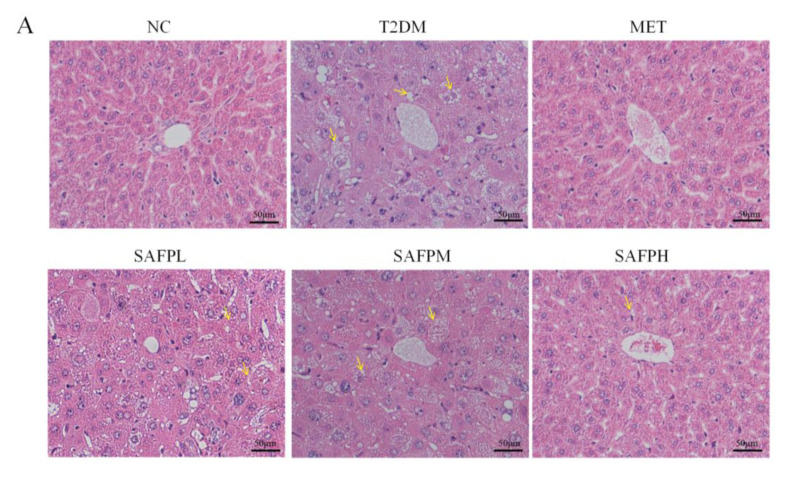

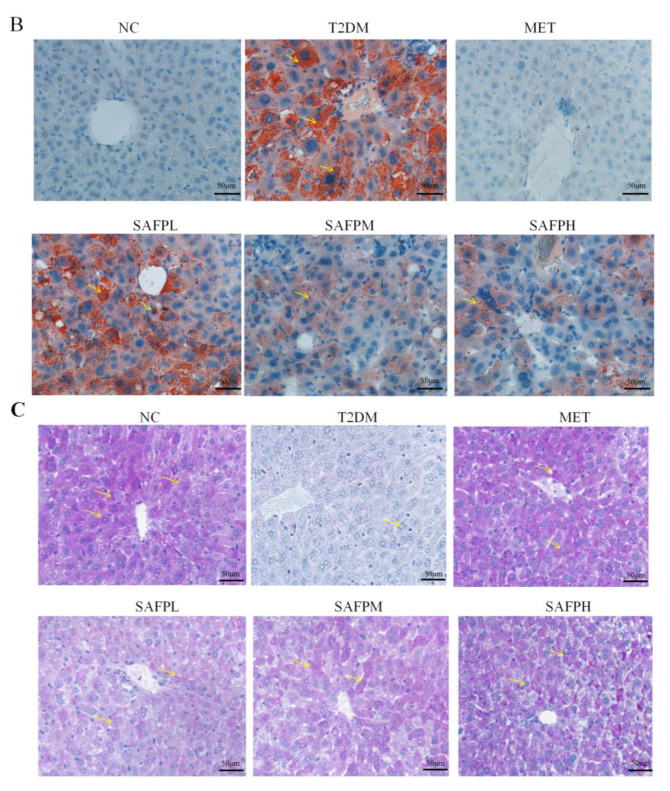
Pathological alterations observed in liver tissue sections. (**A**) H&E staining (scale bars = 50 μm, original magnification: 200×). (**B**) Oil Red O-staining (scale bars = 50 μm, original magnification: 200×). (**C**) PAS staining (bars = 50 µm, original magnifications, 200×).

**Figure 6:**
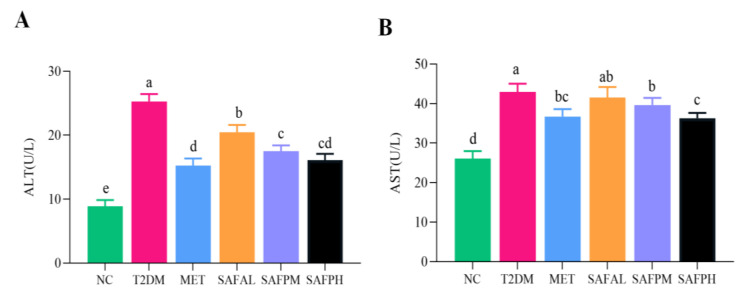
ALT (**A**) and AST (**B**) levels. Values are represented by mean ± SD (*n* = 8). Different small letters indicate that values are significantly different at the *p* < 0.05 level.

As illustrated in Figure 4, the T2DM group exhibited a significant increase in liver weight and hepatic index compared to the NC group, indicating an accumulation of lipids within the hepatic tissue. Surprisingly, the intervention of SAFP reduced liver weight and liver index. Figure 5A reveals that NC group hepatocytes maintained typical morphology with orderly arrangement, devoid of lipid accumulation or inflammatory infiltration. In contrast, the T2DM group displayed swollen hepatocytes accompanied by inflammatory cell infiltration and pronounced steatotic changes. Notably, significant fat vacuolation was observed within the cytoplasm, indicating severe hepatic injury. Remarkably, SAFP supplementation substantially mitigated these pathological manifestations including fatty liver alterations. Oil red O staining further confirmed minimal lipid droplets in NC group livers, while T2DM hepatic sections exhibited extensive lipid deposition that was visibly reduced through SAFP intervention. (Figure 5B). PAS staining was conducted to assess glycogen content in liver tissue of mice. As shown in the representative images of the liver sections, in contrast to the normal mice, the hepatic glycogen content was noticeably decreased in T2DM mice as the liver has an impaired ability to synthesize hepatic glycogen, which was notably elevated after SAFP supplementation, especially at the high dose (400 mg/kg) (Figure 5C). AST and ALT levels are critical indices for evaluating both acute and chronic hepatic injury. Furthermore, as illustrated in Figure 6, the levels of ALT and AST in the T2DM group were significantly elevated compared to those in the NC group. Importantly, these biomarkers of liver injury showed substantial attenuation after SAFP treatment, particularly pronounced at the higher dosage (400 mg/kg) as illustrated in Figure 6. These findings collectively indicated that SAFP intervention effectively mitigated hyperglycemia-associated hepatic damage in T2DM murine models through multiple protective mechanisms.

### 3.4. SAFP Intervention Reduces Liver Oxidative Stress and Inflammation

**Figure 7:**
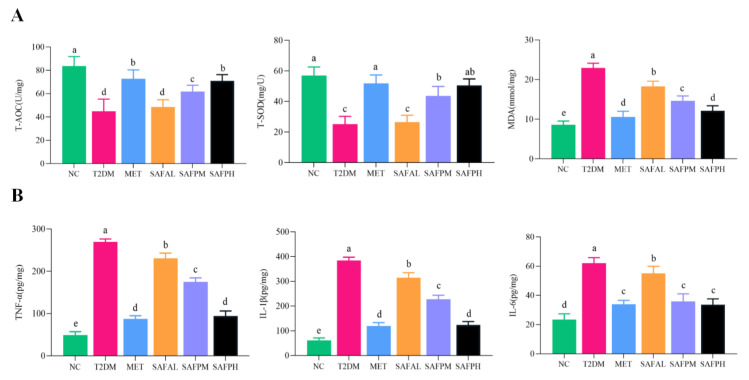
SAFP intervention reduces liver oxidative stress and inflammation. The levels of (**A**) T-AOC, T-SOD and MDA, (**B**) TNF-α, IL-1β, and IL-6 were determined. Values are represented as mean ± SD (*n* = 8). Different small letters indicate that values are significantly different at the *p* < 0.05 level.

As illustrated in Figure 7A, compared with the NC group, hyperglycaemia led to oxidative stress damage on the liver of T2DM mice, as strongly demonstrated by the increase in the level of MDA and the decrease in T-SOD and T-AOC activities. Notably, the higher activities of T-SOD and T-AOC, and the lower MDA level were observed after SAFP administration, when the dosage was 400 mg/kg. Additionally, Figure 6B reveals substantial up-regulation of hepatic pro-inflammatory mediators (TNF-α, IL-1β, IL-6) in diabetic mice. However, treatment with SAFP remarkably down-regulated the expressions of TNF-α, IL-1β and IL-6 in contrast with T2DM group. These results indicated that SAFP intervention could effectively ameliorate hyperglycemia-associated oxidative stress and inflammatory reactions.

### 3.5. Effects of SAFP on Hepatic AMPK Signaling Pathway

**Figure 8:**
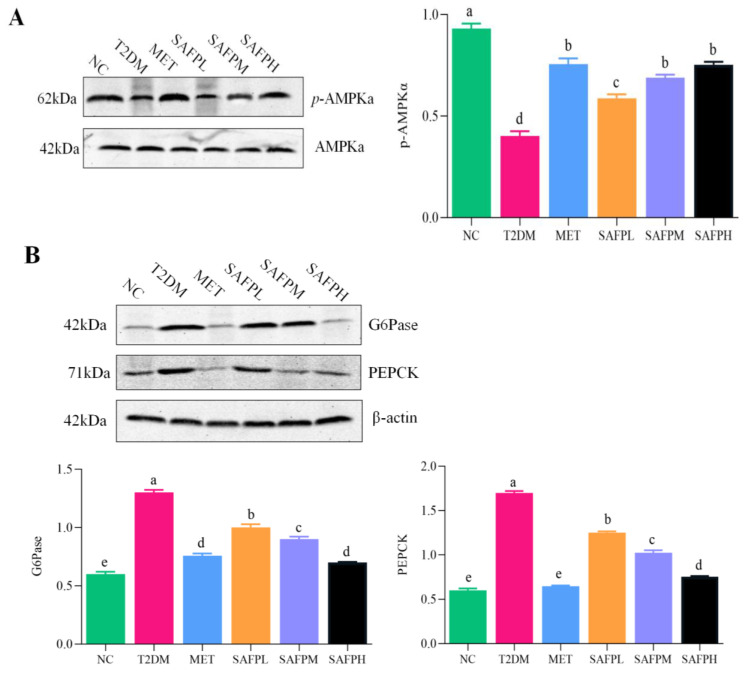
Effects of SAFP on hepatic AMPK signaling pathway. (**A**) Representative Western blotting results and quantitative analyses of p-AMPKα and AMPKα, (**B**) Representative Western blotting results and quantitative analyses of G6Pase and PEPCK across the different groups are presented. β-Actin was employed as a normalization control for protein expression levels. Values are represented by mean ± SD (*n* = 8). Different small letters indicate that values are significantly different at the *p* < 0.05 level.

To assess the impact of SAFP on hepatic glycolipid metabolism, we evaluated the protein expression levels of AMPKα, p-AMPKα, G6Pase, and PEPCK. As shown in Figure 8A, Western blot analysis indicated that compared to the NC group, the expression of hepatic p-AMPKα had significantly decreased. The expression of G6Pase and PEPCK and the expression of hepatic p-AMPKα had significantly decreased, while the expression of G6Pase and PEPCK were markedly elevated. (Figure 8B). Notably, SAFP effectively reversed these changes in a dose-dependent manner, especially since the dose of SAFP was 400 mg/kg. *p*-AMPKα (phosphorylated AMP-activated protein kinase α) represents the active form of AMPKα and can activate the AMPK signaling pathway. Thus, subsequent to the SAFP intervention, there was an up-regulation in the levels of p-AMPKα, leading to the activation of the AMPK pathway, leading to an improvement in glycolipid metabolism dysfunction within the liver. These results demonstrated that SAFP exerted hypoglycemic and hypolipidemic effects by activating AMPK pathway.

### 3.6. SAFP Intervention Enhanced the Intestinal Mechanical Barrier Function

**Figure 9:**
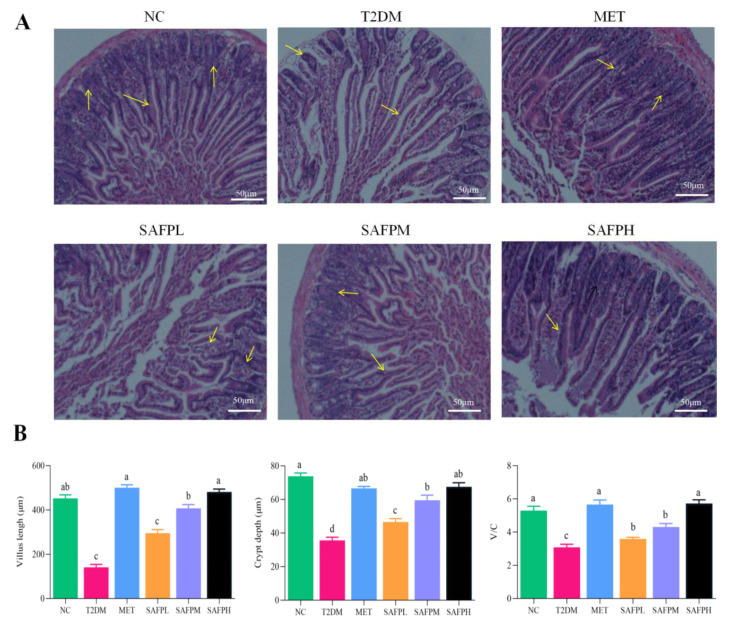

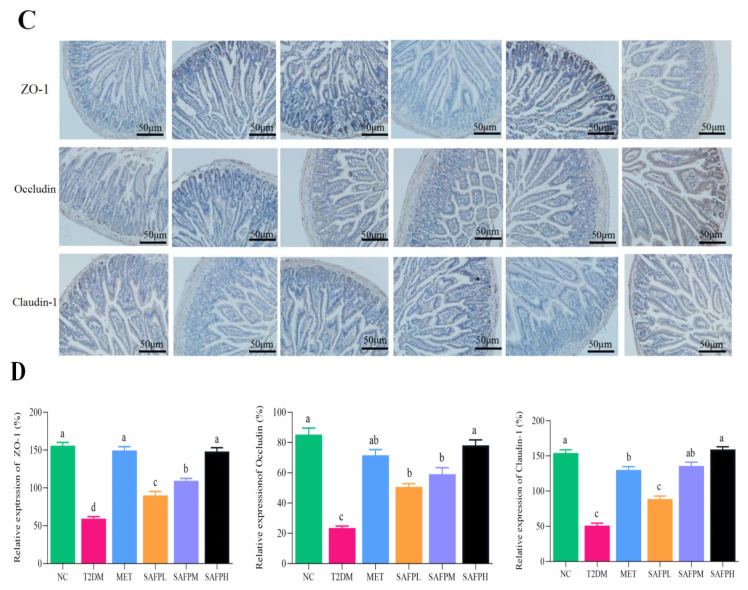
The SAFP intervention significantly enhanced the mechanical barrier function of the intestine. (**A**) Histological examination of the jejunum was conducted following H&E staining in mice (scale bars = 50 µm, original magnification: 200×). (**B**) Measurements included villus length, crypt depth, and the ratio of villus height to crypt depth (**A**/**C**). (**C**) The expression levels of tight junction proteins, including ZO-1, Occludin, and Claudin-1 in the small intestine, were assessed using immunofluorescence staining (scale bars = 50 µm; original magnification: 200×). (**D**) Quantitative analyses of ZO-1, Occludin, and Claudin-1 are presented. Values are expressed as mean ± SD (*n* = 8). Different small letters indicate that values are significantly different at the *p* < 0.05 level.

The effect of SAFP administration on intestinal mechanical barrier was further explored. As indicated in Figure 9A, the H&E staining result of the jejunum sections exhibited an intact physiological structure and intestinal villi were tightly arranged in normal mice. In contrast, as indicated by the yellow arrows in the histological analysis of intestinal tissue, the T2DM group displayed substantial atrophy and fragmentation of the intestinal lumen villi, along with reduced crypt depth and slight edema, which manifested intestinal mucosa damage. Notably, treatment with SAFP led to a significant reversal of this outcome, as evidenced by the reduction in edema and epithelial disruption. Additionally, quantitative analysis (Figure 9B) revealed significant enhancement of villus height, crypt depth expansion, and optimized villus-crypt ratios, all of which exhibited a positive correlation with the dosage of SAFP (Figure 9B). When compared to the NC group, the expression levels of tight junction proteins, including occludin, claudin-1, and ZO-1, were significantly reduced in the T2DM group. Administration of SAFP, particularly at the 400 mg/kg dose, effectively reversed this suppression as evidenced in Figure 9C,D. Therefore, the findings indicated that SAFP supplementation alleviated intestinal pathological changes in T2DM mice, as well as diminished epithelial disruption and intestinal mucosa injury. Additionally, it appears to promote the integrity of the intestinal physiological structure and enhance the intestinal mechanical barrier function, which is achieved by upregulating tight junction protein expression.

### 3.7. SAFP Intervention Reversed Fecal SCFAs Reduction and Microbial Community Imbalance

**Figure 10:**
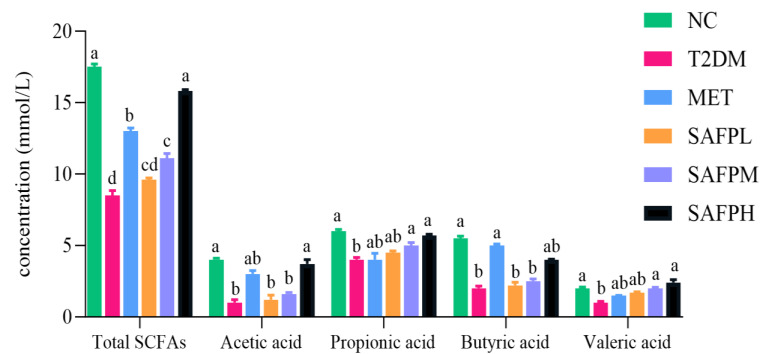
Effects of SAFP on the Concentrations of SCFAs in Feces. Different small letters indicate that values are significantly different at the *p* < 0.05 level.

**Figure 11:**
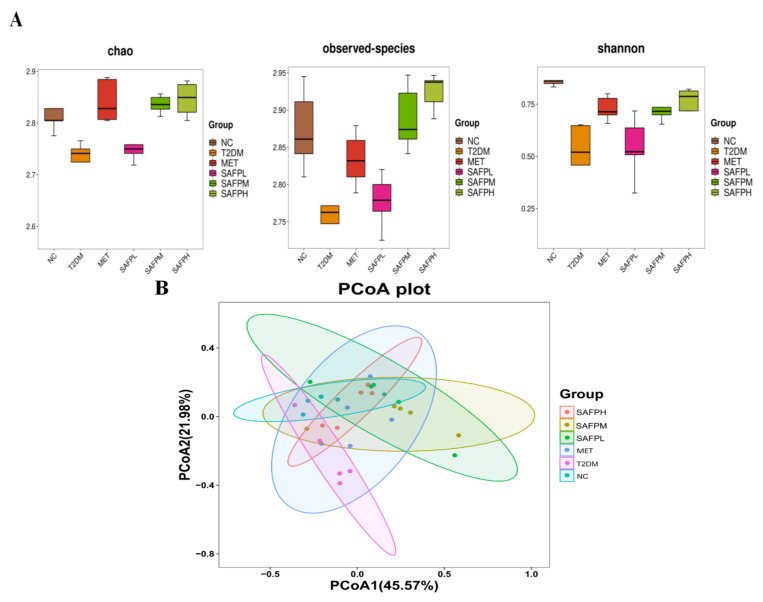
(**A**) Alpha-diversity indices (Chao1, Observed-species and Shannon indices). (**B**) PCoA analysis.

**Figure 12:**
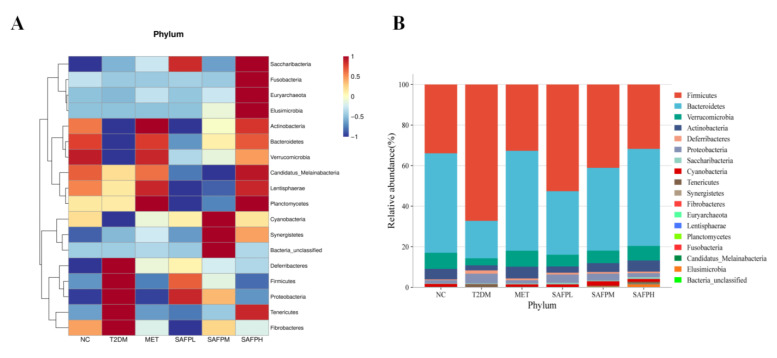

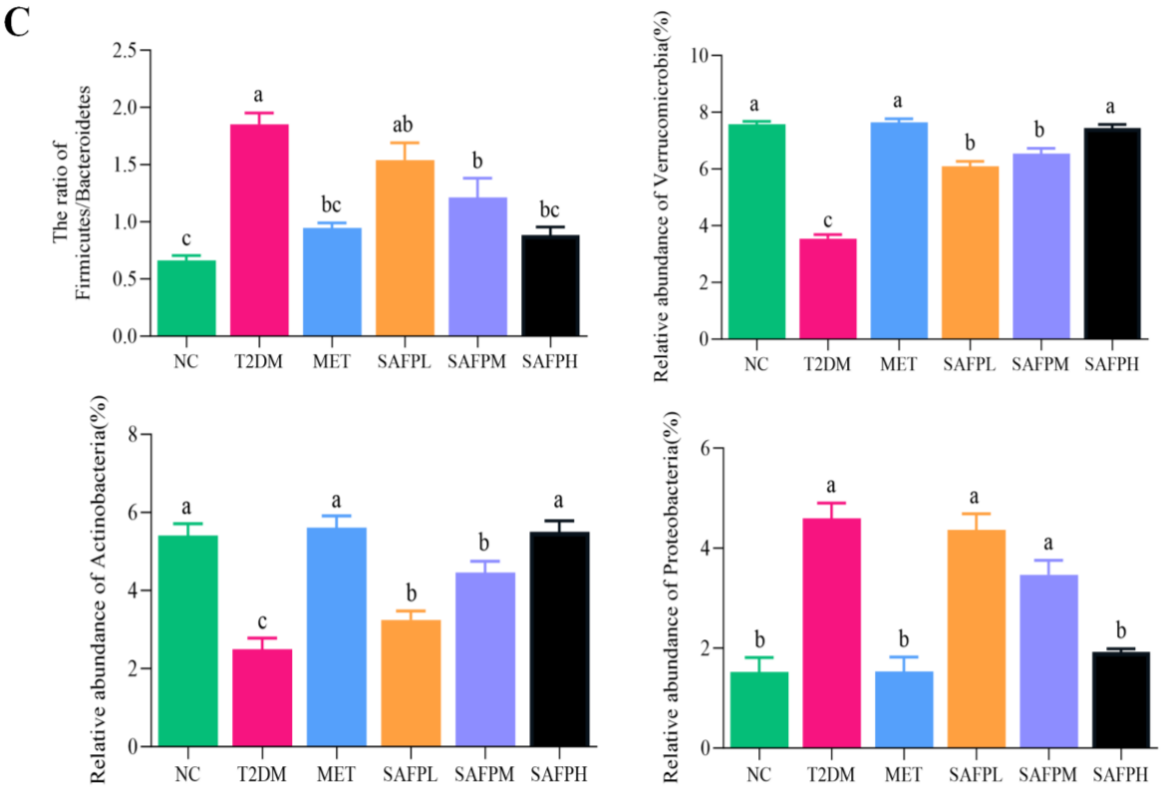
(**A**) Relative abundances of predominant phylum by heatmap analysis. (**B**) Relative abundance of bacteria at the phylum level. (**C**) Relative abundance of *Firmicutes*/*Bacteroidetes* ratio, *Verrucomicrobia*, *Actinobacteria* and *Proteobacteria*. Different small letters indicate that values are significantly different at the *p* < 0.05 level.

**Figure 13:**
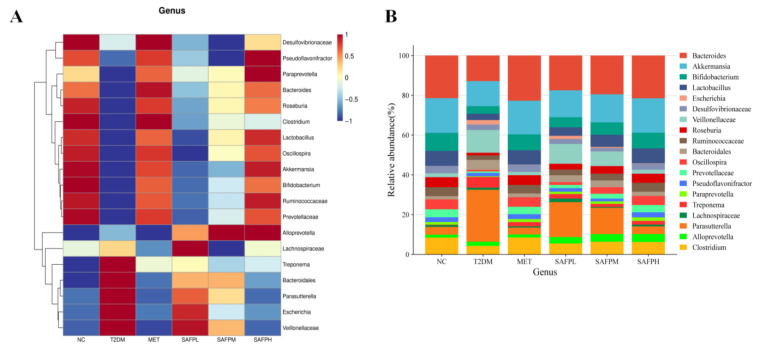

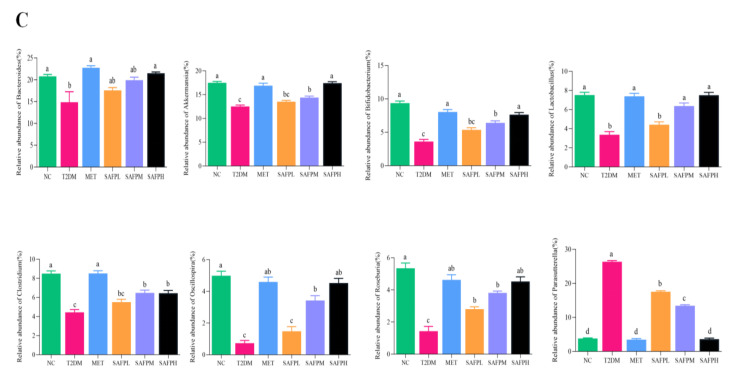
(**A**) Relative abundances of predominant genus by heatmap analysis. (**B**) Relative abundance of bacteria at the genus level. (**C**) Relative abundances of *Bacteroides*, *Akkermansia*, *Bifidobacterium*, *Lactobacillus*, *Clostridium*, *Oscillospira*, *Roseburia* and *Parasutterella*. Different small letters indicate that values are significantly different at the *p* < 0.05 level.

The calibration curves for four short-chain fatty acids (acetic, propionic, butyric, and valeric acids) were detailed in Table 1. As shown in Figure 10, diabetic mice displayed significantly decreased concentrations of acetic acid, propionate acid, butyric acid, valeric acid, and total SCFAs compared to normal control mice. Following SAFP administration, marked elevations were observed in all four SCFA categories, with butyric acid demonstrating the most pronounced enhancement. To investigate intestinal microbial alterations, caecal microbiota composition was examined through 16S rRNA high-throughput sequencing. Figure 11A revealed that diabetic mice exhibited substantially lower alpha-diversity metrics (Chao1, Observed-species, and Shannon indices) relative to healthy controls, suggesting hyperglycemia was associated with diminished microbial richness and diversity. Notably, SAFP treatment effectively counteracted this downward trend in microbial diversity parameters. Figure 11B displayed the gut microbiota’s beta diversity analyzed through PCoA, revealing distinct microbial community variations across experimental groups. Microbiota profiles showed clear separation between T2DM and NC cohorts, with SAFP administration demonstrating substantial modification of intestinal microbial ecosystems in diabetic mice. These findings demonstrated that SAFP effectively boosted bacterial diversity and richness, achieving comparable therapeutic outcomes to MET treatment. Taxonomic profiling combined with heatmap visualization enabled detailed examination of microbial abundance patterns. As depicted in Figure 12A–C, phylum-level analysis exposed marked compositional variations between groups. Compared with controls, T2DM mice exhibited markedly elevated *Firmicutes*-to-*Bacteroidetes* ratios (F/B ratios), which SAFP administration progressively normalized through dose-responsive modulation. Additionally, elevated blood glucose levels were associated with decreased populations of *Verrucomicrobia* and *Actinobacteria* phyla, alongside a marked proliferation of Proteobacteria species. These microbial alterations were ameliorated through SAFP administration. Microbial composition analysis at the genus level (Figure 13A–C) demonstrated significant differences between experimental groups. Compared with NC, T2DM mice exhibited diminished proportions of beneficial genera including *Bacteroides*, *Akkermansia*, *Bifidobacterium*, *Lactobacillus*, *Clostridium*, *Oscillospira*, and *Roseburia*, while displaying elevated *Parasutterella* levels. Crucially, SAFP treatment effectively counteracted these dysbiotic patterns. These findings suggested the therapeutic potential of SAFP in restoring microbial equilibrium through selective enhancement of advantageous bacterial strains and suppression of detrimental microorganisms.

## 4. Discussion

As a multifaceted chronic metabolic disease, the main causes of T2DM are inadequate insulin secretion and insulin resistance. Our experimental protocol involved inducing T2DM in murine subjects through a combination of high-fat dietary regimen and streptozotocin administration. In contrast to the normal mice, weight loss and glycogen metabolism disturbances were observed in diabetic mice, which were reversed after SAFP intervention. We conducted the pre-experiment to ensure that SAFP is non-toxic to mice and demonstrates a high level of safety before we started the formal experiment. In addition, our prior research also demonstrated that SAFP exhibited no toxicity to either cells or mice [18,19]. Our previous research demonstrated that SAFP mainly consisted of D-mannose, D-glucose, D-galactose, and L-fucose with molar ratios of 1.0:5.16:4.75:1.34 [18]. In our previous report, *Cordyceps cicadae* polysaccharides, which also mainly contained D-mannose and D-galactose, improved insulin resistance and glucose tolerance in T2DM rats [22]. Dyslipidemia resulting from lipometabolic disturbance is a significant risk factor for the development of atherosclerosis. Clinical evidence indicates that individuals with hyperlipidemia face heightened cardiovascular risks, particularly developing arterial plaque formation and coronary circulation impairments [27]. As one of the atherogenic lipid indices, elevated levels of LDL-C can contribute to the formation of atherosclerotic plaques that accumulate in the vascular walls [28]. The finding demonstrated that with SAFP supplementation exhibited beneficial effects and relieved the above-mentioned indicators in T2DM mice.

As a crucial metabolic organ that is responsive to insulin, the liver orchestrates glucose and lipid balance by modulating hormonal signals including insulin, glucagon, and catecholamines [29]. Recently, accumulating evidence has suggested that insulin resistance, which may be attributed to energy imbalance, is regarded as the critical pathophysiological characteristic of metabolic syndrome [30,31,32]. This condition facilitates the development of various metabolic diseases including T2DM [33]. Consequently, therapeutic strategies targeting hepatic protection and insulin sensitivity enhancement hold significant potential in T2DM management. Hyperglycemia induces an increase in metabolic activity through mitochondrial glucose oxidation, which typically results in an imbalance between the production of reactive oxygen species (ROS) and the antioxidant defense mechanisms. The abnormal elevation of pro-inflammatory cytokines (such as TNF-α, IL-1βand IL-6) may exacerbate islet dysfunction in individuals with diabetes [34,35]. The excessive generation of ROS rapidly interacts with polyunsaturated fatty acids, leading to the formation of lipid peroxides such as MDA [36]. T-AOC represents the overall antioxidant capacity of the liver, whereas SOD serves as a crucial antioxidant enzyme. Experimental findings revealed that diabetes-induced oxidative stress damage and mild chronic inflammation in the liver. Additionally, hepatic steatosis and impaired liver function were observed in diabetic conditions. Natural polysaccharides such as *Ganoderma lucidum* exhibited anti-inflammatory properties [37]. Our results demonstrated that SAFP treatment markedly ameliorated oxidative stress injury, low-grade chronic inflammation, and hepatic steatosis, indicating that SAFP possessed potent antioxidant and anti-inflammatory properties.

As a conserved serine/threonine kinase and cellular energy balance receptor, AMPK is extensively distributed in tissues characterized by high energy metabolism such as the liver, adipose tissue, and skeletal muscle. AMPK plays a crucial role in the regulation of glucose homeostasis, insulin sensitivity, and cellular energy metabolism [38,39]. In individuals with diabetes, hepatic gluconeogenesis results in an increased hepatic glucose output, which subsequently triggers IR. This phenomenon is recognized as the primary contributor to the abnormal elevation of FBG levels [40,41]. Activated AMPK can downregulate the expression of two critical rate-limiting enzymes, PEPCK and G6Pase, which are associated with hepatic gluconeogenesis. Such regulatory intervention suppresses excessive hepatic glucose generation while curbing endogenous glucose production, thereby contributing to improved fasting glycemic control [42,43]. The AMPK pathway is essential for the regulation of systemic glucose and lipid equilibrium in insulin-resistant rodent models [44,45]. As a result, AMPK is considered a promising therapeutic target for the treatment of diabetes mellitus (DM) [46]. The finding provided compelling evidence that diabetes significantly inhibits hepatic AMPK signaling and disrupts glycolipid homeostasis. Notably, SAFP exhibited hypoglycemic and hypolipidemic effects by activating the hepatic AMPK pathway in T2DM mice.

Recent studies have demonstrated that individuals with T2DM, as well as corresponding animal models, exhibit compromised intestinal barrier integrity [47,48]. This deterioration can facilitate increased absorption of pathogens and toxins within the intestinal lumen, subsequently leading to intestinal inflammation and exacerbation of hyperglycemia [49,50]. Maintaining normal intestinal architecture constitutes the foundation for proper mucosal barrier operation. Intestinal villi and crypts play integral roles in nutrient digestion and absorption, with their dimensional characteristics (villus height and crypt depth) serving as critical parameters for evaluating intestinal structural integrity [4]. Studies have demonstrated that HFD-induced diabetes can lead to alterations in intestinal function, including the down-regulation of intestinal villi height and the up-regulation of crypt depth in mice [51]. As essential components of the intestinal mucosal barrier system, tight junction proteins such as ZO-1, Occludin, and Claudin-1 play a crucial role in maintaining the integrity of the intestinal mucosal barrier by regulating permeability among epithelial cells [52]. The findings supported the conclusion that SAFP intervention enhanced intestinal barrier integrity and intestinal mechanical barrier function by up-regulating the expression of tight junction protein.

Emerging research highlights the vital contribution of gut microbial communities in modulating host nutrient assimilation and metabolic energy processes [53]. Therefore, sustaining microbial balance within the gut ecosystem has emerged as a promising approach to safeguard intestinal barrier function and manage persistent metabolic disorders [54]. Recent investigations reveal that *Verrucomicrobia* and *Actinobacteria* demonstrate significant involvement in glycemic control and metabolic equilibrium maintenance [55]. Higher populations of *Bacteroides*, *Bifidobacterium*, and *Akkermansia* show direct associations with improved intestinal barrier structural soundness and enhanced production of tight junction proteins [56,57,58]. *Lactobacillus* strains facilitate lipid assimilation via bile acid synthesis while simultaneously modulating triglyceride, cholesterol, and blood sugar concentrations. Additionally, these microorganisms exhibit anti-inflammatory properties that alleviate intestinal inflammation and facilitate the repair of the intestinal barrier. Polysaccharides derived from edible fungi are selectively fermented in the gastrointestinal tract into SCFAs, which serve as an energy source for the gut microbiota [59]. Butyrate, a principal SCFA, demonstrates multifaceted biological effects by stimulating mucin secretion and up-regulating tight junction protein expression, thereby preserving mucosal integrity. Additionally, it provides energy for colonic cells and exerts a beneficial effect on intestinal barrier dysfunction as well as inflammatory responses [60,61]. The research indicated that SAFP intervention enhanced butyric acid levels by increasing the abundance of butyric acid-producing bacteria, including *Akkermansia*, *Bifidobacterium*, *Lactobacillus*, *Clostridium, Oscillospira, Roseburia.* Furthermore, clinical studies assessing gut microbiota and metabolic markers in human T2DM subjects will be conducted in our next research.

## 5. Conclusions

In summary, this study revealed that SAFP extracted from Sarcodon aspratus effectively mitigated hyperglycemia, hyperlipidemia, insulin resistance, hepatic steatosis, and inflammatory responses in the liver of type 2 diabetic mice. The therapeutic impacts of SAFP on glucolipid metabolism dysregulation and insulin sensitivity impairment were potentially mediated through AMPK pathway activation. Notably, administration of SAFP elevated concentrations of SCFAs while improving intestinal barrier integrity through enhanced expression of tight junction markers ZO-1, Occludin, and Claudin-1. Concurrently, SAFP treatment modulated gut microbial ecology by suppressing pathogenic bacterial populations and promoting beneficial microbial communities. These findings position SAFP as promising natural prebiotic candidates for addressing both metabolic dysfunctions and intestinal barrier impairments associated with type 2 diabetes mellitus.

## Figures and Tables

**Table 1:** The standard curves equation for the four SCFAs.

SCFAs	Standard Curve Equation	R^2^	Retention Time
Acetic acid	y = 2.7219x + 1.3281	0.9990	6.733
Propionic acid	y = 7.0841x − 43.743	0.9906	8.508
Butyric acid	y = 10.455x − 64.461	0.9905	10.187
Valeric acid	y = 13.039x − 20.315	0.9960	11.738

## Data Availability

Data is contained within the article.

## Abbreviations

The following abbreviations are used in this manuscript:

ALT	Alanine aminotransferase
AMPK	Adenosine 5‘-monophosphate (AMP)-activated protein kinase
AMPKα	AMP-activated protein kinase α
*p*-AMPKα	Phosphorylated AMP-activated protein kinase α
AST	Aspartate aminotransferase
AUC	The area under the curve
DM	Diabetes mellitus
FBG	Fasting blood glucose
FINS	Fasting serum insulin levels
G6Pase	Glucose 6-phosphatase
HDL-C	High density lipoprotein cholesterol
HOMA	Homeostasis model assessment
H&E	Hematoxylin and eosin
IHC	Immunohistochemistry
IL-1β	Interleukin-1β
IL-6	Interleukin-6
IR	Insulin resistance
ISI	Insulin sensitivity index
LDL-C	Low density lipoprotein cholesterol
MET	Metformin
OGTT	The oral glucose tolerance test
OTUs	Operational Taxonomic Units
PAS	Periodic acid-Schiff
PCoA	Principal coordinate analysis
PEPCK	Phosphoenolpyruvate carboxykinase
ROS	Reactive oxygen species
SAFP	Sarcodon aspratus polysaccharides
SCFAs	Short-Chain Fatty Acids
T1DM	Type 1 diabetes
T2DM	Type 2 diabetes mellitus
TBST	Tris-buffered saline Tween-20
TC	Total cholesterol
TG	Triglyceride
TNF-α	tumor necrosis factor-α
